# Guillain–Barré syndrome following COVID‐19 vaccine: A case report

**DOI:** 10.1002/ccr3.6451

**Published:** 2022-10-13

**Authors:** Mohammad Sadegh Fakhari, Leila Poorsaadat, Behnam Mahmoodiyeh

**Affiliations:** ^1^ Student Research Committee Arak University of Medical Sciences Arak Iran; ^2^ Department of Neurology, School of Medicine Arak University of Medical Sciences Arak Iran; ^3^ Department of Anesthesiology, School of Medicine Arak University of Medical Sciences Arak Iran

**Keywords:** case report, COVID‐19, Guillain–Barré syndrome, sinopharm vaccine

## Abstract

Coronavirus disease is a viral infection affecting different organs with various morbidities and mortality. Vaccines are used to control the disease. COVID‐19 vaccines have brought many benefits but their adverse effects should not be ignored. Here, we report a case of Guillain–Barré Syndrome Following Sinopharm COVID‐19 Vaccine.

## Abstract 2

Though vaccination against COVID‐19 have brought many benefits, one should consider various adverse effects of the vaccines, as they might result in severe reactions—like Guillain–Barré Syndrome.

## 1 BACKGROUND

Coronavirus disease (COVID‐19) an infectious disease caused by the SARS‐CoV‐2 virus has spread worldwide since March 2020, becoming one of the most serious causes of mortalities, especially among the elderly and patients with underlying diseases.^1^ This virus can affect various organs including kidney, lung, and liver causing organ failure. Therefore, as a way of controlling the spread of COVID‐19 virus, vaccination is known to be a good strategy.^2^ So far, various types of vaccines of different companies have been developed and approved worldwide.^3^ Along with the variety of benefits of vaccines, the side effects should be considered. COVID‐19 vaccines can have a wide range of side effects including mild and common ones—such as fever and myalgia—to more serious side effects including seizure, life threatening allergic reactions and thrombocytopenia.^2^ Recently, however, some uncommon reactions following COVID‐19 vaccination are being captivate to many researchers. As an example, sudden sensorineural hearing loss has lately been reported as an adverse effect following COVID‐19 second dose vaccination.^4^ Recently, probable association between COVID‐19 vaccines and Guillain–Barré Syndrome (GBS) was reported by several researchers.^5^ GBS is an autoimmune acute inflammatory demyelinating polyneuropathy which is usually evoked by an upper respiratory or gastrointestinal tract infection in two‐thirds of patients, leading to ascending weakness of the limbs.^6^ GBS has been reported following both COVID‐19 infection and its vaccines.^5^, ^7^ The presented case, is one of the first reported cases of GBS after COVID‐19 Sinopharm vaccine, in the absence of any other triggering factors. It is important to be aware of side effects of Sinopharm vaccine in form of GBS to avoid delayed treatment for the patients presenting with the same scenario.

## 2 CASE PRESENTATION

A 60‐year‐old Iranian man appears to Emergency Department (ED) at Valiasr hospital in Arak, complaining of progressive weakness and numbness of his extremities. He had no family history and had a normal psychosocial function. He had a medical history of hypertension and hypothyroidism from years ago and was treated with Levothyroxine, Valsartan, Amlodipine, and Aspirin in advance. He reported a 3 weeks history of mild paresthesia of his fingertips which developed into weakness of both upper and lower distal extremities during this time. There was no recent history of fever, cough, shortness of breath, nausea, vomiting, diarrhea, and there was no recent travel history. His family members were self‐isolating and had no symptoms of COVID‐19. He reported a history of taking three doses of Sinopharm Vaccine and the third dose was administered about 20‐days before the onset of his symptoms.

On admission to ED, he was afebrile, his blood pressure was 140/90 mmHg with a pulse rate of 90 bpm, and normal oxygen saturation of 98% on room air. Neurologic physical examinations (including orientation, memory, cranial nerves, and cerebellar tests) were normal, apart from absent deep tendon reflexes (DTR) of lower limbs and reduced force of proximal and distal of his lower limbs. Examinations showed normal DTR and force of upper limb, normal muscle tone, and negative Babinski sign. There was no meningism, spinal cord sensory level, bladder, or bowel involvement.

## 3 INVESTIGATIONS

Laboratory tests including complete blood count, electrolytes, creatinine, liver function tests were all normal, except for an Erythrocyte Sedimentation Rate of 36 mm/hour (reference range < 10), a one‐unit positive C‐reactive protein, and creatine phosphokinase of 695 units/liter (reference range: 24–195). Coombs Wright and 2‐mercaptoethanol were both negative for Brucellosis. Chest CT scan findings were normal, except for atelectasis in lung bases and degenerative joint disease of the spine. MRI of the brain, cervical, thoracic, and lumbar Spine was reported normal. Echocardiography and electrocardiography (ECG) did not show any pathologic finding. Lumbar puncture was not performed due to the patient's discontent. His neurophysiology study showed low amplitude sensory response and reduced amplitude of motor response in lower limb without any decrease in velocity. No abnormalities were observed in his electromyography since we had performed the test prior to development of the symptoms, during the first week after admission. Meeting five of seven domains of Brighton Criteria,^8^ diagnosis of GBS was confirmed, and the report of neurophysiology study is with acute motor‐sensory axonal neuropathy (AMSAN) variant of GBS. Tables 1 and 2 demonstrate the neurophysiology study results. (Tables 1, 2).

**TABLE 1:** Neurophysiology results for the **Lower Limb**

A. Motor Nerve Conduction Studies (NCS)			F‐Wave Latency (ms)		Latency (ms)		Amplitude (μV)		Conduction Velocity (m/s)
Right Peroneal	Distal		38 (≤56)		5.6 (≤6.5)		0.9 (≥2)		44 (44)
	Proximal				13.8 (≤6.7)		1.4 (≥5)		
Right Tibial	Distal		‐		4.9 (≤6.3)		5.1 (≥3)		46 (41)
	Proximal				15.4 (≤5.8)		1.9 (≥4)		
Left Peroneal	Distal		49 (≤56)		3.8 (≤6.5)		1.3 (≥2)		48 (44)
	Proximal				14.2 (≤6.7)		0.5 (≥5)		
Left Tibial	Distal		‐		5.2 (≤6.3)		6.3 (≥3)		49 (41)
	Proximal				12.9 (≤5.8)		5.2 (≥4)		
**B. Sensory NCS**									
Right Sural			‐		3.4 (≤4.4)		4 (≥6)		40 (≥40)
Left Sural			‐		4 (≤4.4)		3 (≥6)		40 (≥40)
**C. Needle Electromyography**									
Muscle	Spontaneous activity				Motor units				Recruitment
	Insertion	Fib	PSW	Fasc	Firing	Amp	Dur	Poly.	Pattern
Anterior Tibialis	None	None	None	None	3+	Normal	Normal	None	Discrete
Right gastrocnemius	None	None	None	None	3+	Normal	Normal	None	Discrete
Left gastrocnemius	None	None	None	None	3+	Normal	Normal	None	Discrete
Vastus Lateralis	None	None	None	None	3+	Normal	Normal	None	Discrete
Interpretation	Can be prolonged in denervation	Few Fibs and PSW can be normal but profuse in complete denervation		Can be present in normal muscle denervation	Rapid firing in denervation	Can be normal in acute nerve lesions			Full interference in healthy muscle Discrete recruitment in denervation as more motor units drop out

**TABLE 2:** Neurophysiology results for the **Upper Limb**

A. Motor Nerve Conduction Studies (NCS)						Latency (ms)		Amplitude (μV)	Conduction Velocity (m/s)
Right Median		Distal				4 (≤4.4)		4.3 (≥4)	55 (≥49)
		Proximal				8.9 (≤4.4)		4.9 (≥4)	
Right Ulnar		Distal				3.2 (≤3.3)		8.2 (≥6)	59 (≥49)
		Proximal				7.8 (≤4.5)		8 (≥7)	
Left Median		Distal				3.7 (≤4.4)		4.1 (≥4)	60 (≥49)
		Proximal				8.5 (≤4.4)		3.9 (≥4)	
**B. Sensory NCS**									
Right Median						2.1 (≤3.5)		24 (≥20)	60 (≥50)
Right Ulnar						1.9 (≤3.1)		26 (≥17)	55 (≥50)
Left Median						2 (≤3.5)		25 (≥20)	63 (≥50)
**C. Needle Electromyography**									
Muscle	Spontaneous activity				Motor units				Recruitment
	Insertion	Fib	PSW	Fasc	Firing	Amp	Dur	Poly.	Pattern
Right Flexor Carpi Ulnaris	None	None	None	None	3+	Normal	Normal	None	Discrete
Left Flexor Carpi Ulnaris	None	None	None	None	3+	Normal	Normal	None	Discrete
Right Biceps Brachii	None	None	None	None	3+	Normal	Normal	None	Discrete
Left Biceps Brachii	None	None	None	None	3+	Normal	Normal	None	Discrete
Interpretation	Can be prolonged in denervation	Few Fibs and PSW can be normal but profuse in complete denervation		Can be present in normal muscle denervation	Rapid firing in denervation	Can be normal in acute nerve lesions			Full interference in healthy muscle Discrete recruitment in denervation as more motor units drop out

## 4 TREATMENT

The patient was monitored in intensive care unit (ICU) for the first 3 days of hospitalization and did not show any respiratory or swallowing dysfunction. He received intravenous immunoglobulin (IVIG) 0.4 g/kg daily for 5 days along with prophylaxis Heparin and Pantoprazole therapy and physiotherapy of his extremities. His symptoms subsided and were stabilized during hospitalization, and he did not require intubation.

## 5 OUTCOME AND FOLLOW‐UP

The patient's motor symptoms began to improve subsequently to the first days of treating with IVIG. In 3 days after ICU admission, his signs and symptoms eased up and he was transferred to ward until his treatment was completed and at the sixth day of hospitalization, he was able to walk unassisted and was discharged on the seventh day.

## 6 DISCUSSION

Due to severe consequences, it is vital to diagnose and treat GBS with no delay. Symptoms such as paresthesia and difficulty in moving after COVID‐19 vaccination should not be missed, since there have been several reports of patients presenting with the same symptoms after vaccination. GBS has been reported to occur following various types of COVID‐19 vaccines so far,^9^ however, to the author's awareness, GBS symptoms following Sinopharm COVID‐19 vaccine have been reported infrequently.

Sinopharm COVID‐19 vaccine presents a dead copy of the virus to the body by a two‐dose schedule, followed by a third booster dose after a period of at least 3 months. Dead antigens of the virus introduced to the immune system, bring about antibodies and prepare the immune system for further attacks by virus. In August 2020, trials of this vaccine were completed and showed activation of neutralizing antibody response as a result of vaccine injection with low rates of adverse reactions. The most common adverse effects were pain at the injection site and fever which were all mild and required no treatment.^10^

Confirming post‐vaccination GBS diagnosis, requires the absence of other etiologies and beginning symptoms within 6 weeks after receiving the vaccine.^11^ The pathophysiological mechanism of post‐vaccination and post‐infectious GBS is thought to be the same, as a delayed immune‐mediated reaction unveiled by the activation of T‐cells which cross‐react to both viral antigen and a myelin protein, causing clinical manifestations of GBS.^12^, ^13^

Our case did not report any symptoms nor did our investigations raise any doubt about the etiology of GBS except for a history of Sinopharm COVID‐19 vaccination. Meticulous post‐vaccination observation and reporting system would clarify the relationship between different types of COVID‐19 vaccines and GBS.

This report should be evaluated in light of its strength and limitations. It should be noted that our case was immediately and carefully managed, and he was satisfied with the personnel's behavior and management. However, some limitations such as distraction of reader when focusing on the unusual and the retrospective design are limitations of this study which are inherent to this type of publication.

## 7 CONCLUSION

Herein, we reported a case of an old male having GBS following Sinopharm COVID‐19 vaccine. It should be noted that due to severe consequences of GBS, the condition must be considered following COVID‐19 vaccination.

## AUTHOR CONTRIBUTIONS

MSF, LP, and BM helped in study conception, design, data collection, statistical expertise, analysis, and interpretation of data. MSF and LP contributed to manuscript preparation, supervision, administrative support, and critical revision of the paper. All authors read and approved the final manuscript.

## FUNDING INFORMATION

This article was no funded by any individual or organization.

## CONFLICT OF INTEREST

All the authors declared no conflict of interest.

## ETHICAL APPROVAL AND CONSENT TO PARTICIPATE

A written informed consent was obtained from the participant. The authors confirm that all methods were performed in accordance with institutional ethical standards and the Declaration of Helsinki.

## CONSENT

A written informed consent for publicly reporting the information of the condition was obtained from the participant.

## Data Availability

All data are available from the corresponding author on reasonable request.
